# The Relationship between Serum Carbonic Anhydrase I-II Autoantibody Levels and Diabetic Retinopathy in Type 1 Diabetes Patients

**DOI:** 10.4274/tjo.99233

**Published:** 2017-04-01

**Authors:** Adem Türk, Süleyman Mollamehmetoğlu, Ahmet Alver, Ahmet Menteşe, İrfan Nuhoğlu, Cihangir Erem, Halil İbrahim İmamoğlu

**Affiliations:** 1 Karadeniz Technical University Faculty of Medicine, Department of Ophthalmology, Trabzon, Turkey; 2 Of State Hospital, Ophthalmology Clinic, Trabzon, Turkey; 3 Karadeniz Technical University Faculty of Medicine, Department of Biochemistry, Trabzon, Turkey; 4 Karadeniz Technical University Faculty of Medicine, Department of Internal Medicine, Trabzon, Turkey

**Keywords:** Carbonic anhydrase I, carbonic anhydrase II, diabetes mellitus, diabetic retinopathy

## Abstract

**Objectives:**

To investigate the relationship between serum carbonic anhydrase I-II (CA-I and II) autoantibody levels and diabetic retinopathy (DRP) in cases with type 1 diabetes.

**Materials and Methods:**

A total of 37 type-1 diabetic patients, 17 with DRP (group 1) and 20 without (group 2), and 38 healthy control subjects (group 3) were included. CA-I and CA-II autoantibody levels were measured in serum samples obtained from each of the three groups and compared statistically. Additionally, the correlation between CA-I and CA-II autoantibody levels and the presence of diabetic macular edema was examined.

**Results:**

Mean measured CA-I autoantibody levels were 0.145±0.072, 0.117±0.047, and 0.138±0.061 ABSU in group 1, group 2, and group 3, respectively (p=0.327). The average CA-II autoantibody levels achieved in the same groups were 0.253±0.174, 0.155±0.137, and 0.131±0.085 ABSU, respectively (p=0.005). No significant difference was obtained between the subgroups of group 1, with macular edema (n=8) and without (n=9), in terms of both CA-I and CA-II autoantibody levels (p=0.501, p=0.178, respectively).

**Conclusion:**

A significant correlation was observed between the development of DRP and serum CA-II autoantibody levels in type 1 diabetic cases. However, there was no correlation between the autoantibody levels and the presence of diabetic macular edema in cases with DRP.

## INTRODUCTION

Diabetes mellitus is a chronic endocrine disease which is common worldwide and can cause various micro- and macrovascular complications. Diabetic retinopathy (DRP), an important microvascular complication of the disease, is a common cause of vision loss.^1,2^

Type 1 diabetes, one of the subtypes of diabetes mellitus, is becoming a major health problem as its prevalence steadily rises, especially in the younger population. Type 1 diabetes develops as a result of destruction of pancreatic beta cells due to genetic and multifactorial immune response. Islet cell antibodies were detected in the human pancreas for the first time in the 1970s. In subsequent decades, the presence of various autoantibodies such as glutamic acid decarboxylase antibody, microinsulin antibody, and zinc transporter antibody were reported.^3,4,5^

The carbonic anhydrases (CA), members of the zinc metalloprotein family, are enzymes which catalyze the interconversion of carbon dioxide and water to bicarbonate and hydrogen ions. To date, about 16 CA isoenzymes responsible for various functions have been identified.^6^ The CA isoenzymes found in the eye also serve different functions based on their location.^7^ CA-I has been found in corneal endothelial cells, lens cells, capillary endothelial cells, in the stroma of the ciliary body, and in the choroid.^8^ CA-II has been documented in the ciliary body epithelial cells, retinal Müller cells, the retinal pigment epithelium, cone photoreceptors, and the choriocapillaris.^8,9,10,11,12,13,14,15,16^

Various studies have reported that autoantibodies to CA isoenzymes increase during the course of some immunological diseases.^17,18,19,20,21^ Based on this premise, the aim of our study was to investigate the presence of CA autoantibodies in patients with type 1 diabetes, which is considered an immunological disease, and to examine possible associations between these autoantibodies and DRP.

## MATERIALS AND METHODS

This cross-sectional study was approved by the Ethics Committee and all participants provided informed consent. Thirty-seven type 1 diabetes patients between 13 and 58 years of age and 38 healthy control subjects between 23 and 54 years of age were included in the evaluations.

### Evaluation Criteria

The diabetic patients selected for the study were recruited in part from type 1 diabetes patients being followed in the retina division, and in part from type 1 diabetes patients presenting to the ophthalmology outpatient clinic for examination. Patients with Sjögren’s syndrome, autoimmune hepatitis, primary biliary cirrhosis, and other autoimmune conditions such as Graves’ disease were not included. Patients with history of uveitis or glaucoma were also not included. Patients underwent a thorough ophthalmic examination upon being accepted to the study. After biomicroscopic examination of the anterior and posterior segments, mydriatic eye drops were instilled in both eyes. After pupil dilation, fundus photographs were taken and optical coherence tomography (OCT) images were obtained to evaluate whether macular edema was present. Based on the examination findings, the patients were divided into two groups: those with DRP findings (group 1) and those without (group 2).

The main criterion for inclusion as a healthy control subject (group 3) was having no systemic or ocular problems other than refractive error.

### Collection and Analysis of Blood Specimens

Venous blood was collected into biochemical tubes once from all subjects in each of the three groups. After 5 minutes at room temperature, the blood specimens were centrifuged and stored at -80 ˚C. All of the collected specimens were assayed in the biochemistry laboratory during the same time frame using the ELISA method to measure CA-I and CA-II autoantibody levels.

### Statistical Analysis

SPSS version 13.0.1 (SPSS, Chicago, Illinois, USA; License no: 9069728, KTU, Trabzon, Turkey) was used for statistical analyses. Data obtained from the study groups were expressed as mean ± standard deviation (SD). Normal distribution of numerical data was analyzed using the one-sample Kolmogorov-Smirnov test. The one-way ANOVA (post hoc Tukey test) was used in comparisons of quantitative values and the chi-square test was used in comparison of qualitative values from the three groups. The Mann-Whitney U test was used to compare quantitative values from cases in group 1 with and without diabetic macular edema. The relationship between hemoglobin A1c (HbA1c) levels and autoantibody levels in diabetic patients was examined using Pearson correlation analysis, while relationships between autoantibody levels in all study groups were examined by Spearman correlation analysis. Furthermore, independent samples t-test was used to compare CA-I and CA-II autoantibody levels between all of the diabetic patients in groups 1 and 2 (n=37) and the healthy subjects in group 3 (n=38). P values ≤0.05 were accepted as statistically significant.

## RESULTS

Mean ages of the participants were 38.82±8.85 (24-58) years in group 1, 29.75±10.71 (13-54) years in group 2, and 35.58±9.44 (23-54) years in group 3 (p=0.017). There was a significant age difference between groups 1 and 2 (p=0.016), while the age differences between groups 1 and 3 (p=0.081) and groups 2 and 3 (p=0.486) were not significant. Nine of the 17 patients in group 1 were female, 8 of the 20 patients in group 2 were female, and 16 of the 38 healthy volunteers in group 3 were female. There were no significant sex differences between the groups (p=0.692).

Mean duration of diabetes among the diabetic patients in the study was 18.06±7.8 (6-37) years for group 1 and 9.23±8.6 (1-34) years for group 2 (p=0.003). Mean HbA1c levels were 9.4±2.3% (6-14%) in group 1 and 8.8±2% (7-15%) in group 2 (p=0.387).

**Figure 1:**
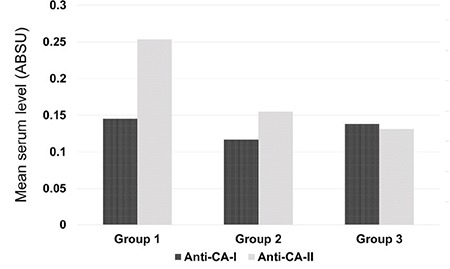
Comparison of serum carbonic anhydrase I and II autoantibody levels in type I diabetes patients with diabetic retinopathy (group 1) and without diabetic retinopathy (group 2), and in the healthy control group (group 3) CA: carbonic anhydrases

Mean CA-I autoantibody levels in groups 1, 2, and 3 were 0.145±0.072, 0.117±0.047, and 0.138±0.061 absorbance units (ABSU), respectively (p=0.327). Mean CA-II autoantibody levels in the same groups were 0.253±0.174, 0.155±0.137, and 0.131±0.085 ABSU (p=0.005) (Figure 1). There was a significant difference in CA-II autoantibody level between groups 1 and 2 (p=0.05) and groups 1 and 3 (p=0.003), while the difference between groups 2 and 3 was not significant (p=0.756).

No significant differences in CA-I or CA-II were found between patients in group 1 who had macular edema on OCT (n=8) and those who did not (n=9) (p=0.501 and p=0.178, respectively).

There was no significant association between HbA1c levels and CA-I autoantibody levels in the diabetic patients in groups 1 and 2 (r=0.12, p=0.445). A weak correlation was detected between diabetic patients’ HbA1c and CA-II autoantibody levels (r=0.36, p=0.027). There was no statistically significant relationship between the CA-I and CA-II autoantibody levels of subjects in the three study groups (n=75) (r=0.146, p=0.212).

Mean CA-I autoantibody levels of all diabetic patients in the study (n=37) and the healthy subjects in group 3 (n=38) were 0.13±0.06 and 0.138±0.061 ABSU, respectively; mean CA-II autoantibody levels in the same groups were 0.2±0.161 and 0.131±0.085 ABSU. Although the difference in CA-I autoantibody level between the diabetic and nondiabetic subjects was not statistically significant (p=0.578), there was a significant difference in CA-II autoantibody level (p=0.023).

## DISCUSSION

The immune system comprises specialized cells that protect an organism’s body from infection and external agents. Under normal conditions, the immune system does not respond to antigens within the organism. This is defined as selective non-responsiveness to antigens. When this tolerance system is disrupted, the immune system becomes unable to differentiate between foreign and host antigenic structures. This results in an autoimmune response triggered by autoantigenic recognition and subsequent autoimmune disease.^22,23^ A search of the literature yields many studies reporting autoimmunity to CA isoenzymes in various autoimmune diseases. The presence of CA autoantibodies has been documented in acute anterior uveitis, Graves’ disease, systemic lupus erythematosus, Sjögren’s syndrome, endometriosis, idiopathic chronic pancreatitis, primary biliary cirrhosis, tubulointerstitial nephritis, autoimmune hepatitis, and autoimmune cholangitis.^17,18,19,20,21,24,25,26,27^

Taniguchi et al.^20,28^ investigated the presence of CA autoantibodies in the etiopathogenesis of type 1 diabetes in two separate studies. In both of the studies, CA-II autoantibody levels were measured in type 1 and 2 diabetes patients, and CA-II autoantibody levels were higher in patients with type 1 diabetes compared to those with type 2 diabetes.^20,28^ The results of those studies supports that type 1 diabetes is an autoimmune endocrinopathy. In the present study, which included only type 1 diabetes patients, both CA-II and CA-I autoantibody levels were compared with those of a healthy control group. Our results that CA-II autoantibody levels are higher in diabetic patients than in the healthy controls corroborate those of Taniguchi et al.^20,28^ However, unlike the studies by Taniguchi et al.,^20,28^ we also investigated the association between CA-II autoantibody levels and the presence of DRP. This analysis revealed that CA-II autoantibody levels in type 1 diabetes patients with DRP were significantly different than those in type 1 diabetes patients without DRP and those of healthy controls. Di Cesare et al.^29^ reported that type 1 diabetic patients showed no significant differences in CA-II autoantibody levels compared to a healthy control group. However, the fact that DRP was not investigated in their studies may explain why the results of Taniguchi et al.^20,28^ and Di Cesare et al.^29^ differ from ours.

In our study, the increase in CA-II autoantibody levels in type 1 diabetes patients was more pronounced in the presence of DRP. Adamus and Karren^30^ reported that CA-II autoantibodies may inhibit CA-II enzyme activity in the retina, which decreases the intracellular pH and leads to the accumulation of intracellular calcium, ultimately resulting in retinal cell dysfunction. This suggests that CA-II autoantibodies may play a role in the etiopathogenesis of both type 1 diabetes and the retinopathy associated with type 1 diabetes. Our finding that CA-II autoantibody levels are statistically elevated in type 1 diabetes patients with DRP further supports this hypothesis.

Levels of CA-I antibody were also evaluated in the present study. There were no significant differences in CA-I levels in type 1 diabetes or in the presence of diabetic retinopathy.

There are no previous studies in the literature examining the relationship between serum CA-I and CA-II levels and the development of DRP in patients with type 1 diabetes. The results of our novel study indicate that CA-I autoantibodies are not significantly associated with the development of type 1 diabetes or DRP. CA-II autoantibodies were significantly elevated only in patients with DRP. The presence of autoantibodies to the CA-II isoenzyme, which functions in many parts of the retina and in the choriocapillaris, may lead to pathologic retinal changes (i.e. DRP) secondary to CA-II dysfunction.

## CONCLUSION

Our study included a small number of patients, which may have influenced the correlations between the analyzed parameters. Therefore, future studies with larger populations are needed to better elucidate these relationships.
